# Widowhood and Life Satisfaction Among Chinese Elderly Adults: The Influences of Lifestyles and Number of Children

**DOI:** 10.3389/fpubh.2021.754681

**Published:** 2022-01-26

**Authors:** Caiyun Yang, Xixi Sun, Wenjie Duan

**Affiliations:** ^1^School of Philosophy and Law & Political Science, Shanghai Normal University, Shanghai, China; ^2^School of Social and Public Administration, East China University of Science and Technology, Shanghai, China

**Keywords:** widowhood status, lifestyle, life satisfaction, number of children, self-regulation theory, moderated mediation

## Abstract

Our study examined how lifestyle and number of children influence the relationship between widowhood and life satisfaction based on self-regulation theory. A sample of 2,968 elderly respondents (male = 1,515, female = 1,453, mean age = 69.12 years, *SD* = 7.24) participated in Chinese General Social Survey. Our findings suggest that lifestyle is positively related to life satisfaction, and number of children is positively associated with life satisfaction but negatively related to lifestyle. The moderated mediation model demonstrated that lifestyle partly mediated the relationship between widowhood and life satisfaction. Moreover, number of children moderated the relationship between widowhood and lifestyle and between lifestyle and satisfaction with life. Widowed elderly individuals who have more children are likely to show a higher level of satisfaction with life. The present study has significance in practice because it provides empirical implications obtained from a national survey on the universal two-child policy in China as two children might decrease the negative impacts of widowhood on life satisfaction.

## Introduction

The possibility of death in married couples increases as they age. According to a census conducted in 2000, the percentage of widowed elderly women in the United Kingdom, Italy, Finland, France, Greece, Australia, and Russia ranged from 52.8 to 65% (1). The Sixth National Population Census of China demonstrated that the widowed elderly adult population was estimated at 47.74 million, which accounted for 26.89% of the elderly adult population in China (2). The widowed population is expected to reach 118.4 million in 2050 as a result of the development of an aging society (3). Previous empirical studies recognized the death of a spouse and bereavement as a major form of trauma among elderly adults (4–6) that negatively influences their physical and mental health, thereby resulting in chronic diseases, disability, dementia, depression (7, 8), and reduced satisfaction with life (9, 10).

Similar studies indicated that widowhood tends to have negative effects on satisfaction with life (11, 12); however, some of these studies further implied that the negative influence may be transient (13). In other words, an individual only temporarily experiences low-level satisfaction with life after losing their spouse; their life satisfaction soon returns to its initial level. Wilder (14) interviewed 23 widowed people, including six widowers and 17 widows; this study found that involvement in a social network, such as maintaining connections with friends and keeping an active lifestyle, tended to help them regain life satisfaction. In other words, individuals might adopt specific lifestyles to maintain their physical and mental well-being (15), and construct new social identities (16). According to the self-regulation model of selection, optimization, and compensation and resilience theory (17), adjusting lifestyles to reconstruct daily life can be recognized as the optimal use of personal assets to handle threatening situations (e.g., losing a spouse). The use of self-regulatory processes, or what are referred to in the self-regulation model as life management strategies, not only facilitates adjustment to loss and limitation, but also may bring about growth and enhance well-being (18, 19).

Previous studies demonstrated that the lifestyle of widowed elderly adults significantly differs from non-widowed ones (20, 21). Once a spouse passes away, the social participation and interaction of an individual narrows (21), thereby depriving them of many social roles (e.g., wives or husbands). Fortunately, some of them might successfully change their lifestyles (e.g., participating in a social network actively and performing increased indoor and outdoor activities) to acclimatize themselves to widowhood (4, 14, 22). Carr and Utz (22) conducted the longitudinal Changing Lives of Elderly Couples study of 316 elderly people who had lost their spouses. The results indicated that changing their lifestyle in several aspects (e.g., social activities, interpersonal relationships, and hobbies) may help widowed elderly people recover from pain. In a sample of 228 widowed elderly adults, a previous study indicated that some elderly people became inclined to positive lifestyle changes after experiencing widowhood (i.e., higher level of informal social participation), thereby enhancing their late-life well-being (23). Thus, we can reasonably hypothesize the mediational role of lifestyle in the relationship of marriage status and life satisfaction.

The various constructions of lifestyle may lead to mixed results. Ha and Ingersoll-Dayton (24) solely focused on social contacts with friends and relatives with one item. They found that contact had no significant effect on improving life satisfaction of widowed elderly people; they used an analytic sample of 209 elderly adults who experienced spousal loss. A lifestyle intervention study for widowed elderly adults demonstrated that widowed elderly people tended to show fewer depression symptoms and higher level of satisfaction after the implementation of a technology-based healthy lifestyle intervention (25). Therefore, Fratiglioni et al. (26) proposed a multidimensional construct of lifestyle, which contained social network (e.g., meeting with relatives who are not living together, gathering with friends), leisure (e.g., listening to music, reading books or magazines, and surfing the internet) and physical or outdoor (e.g., doing physical exercise) activities. The present study aimed to establish a mediation model using a multifaceted lifestyle construct.

China's one-child policy, which was launched in 1979, controlled the number of children in families (27); this policy significantly influenced the lifestyle of elderly adults in Chinese culture. Previous research demonstrated that elderly individuals with one or more children received intergenerational support and had better living conditions compared to those without children (28). Using data from 2012 Chinese General Social Survey (CGSS), Wang and Yuan (29) demonstrated that elderly individuals with one or more children tended to take part in leisure activities more often. In addition, elderly individuals had shown a desire to adjust their lifestyle (e.g., going shopping, attending cultural activities, or watching live sports) to better communicate with their children (30) and foster relationships with younger family members (31). In other words, lifestyle of elderly individuals tends to change because of their children. In addition, life satisfaction could also be influenced by the number of children individuals have. For example, using the 2013 China Health and Retirement Survey data, previous studies indicated that the number of married children positively affected the life satisfaction of elderly parents (32). Moreover, elderly individuals with more children were more likely to enjoy a higher quality of life and better health conditions because they received more health care (33). However, a previous study showed that the relationship between the subjective well-being of elderly adults and the number of their children was not simply linear, but an inverted U-shaped relationship (34). In other words, to a certain extent, the subjective well-being of elderly adults increases with the number of children, and then as the number of children increases, their subjective well-being begins to decline. Fewer children will lead to a high cost of care borne by the children, which is not conducive to the welfare improvement of elder adults. Conversely, too many children will lead to high friction costs in decision making for elderly adults, resulting in a welfare decline. In short, we can hypothesize that the number of children affects lifestyle and life satisfaction among elderly adults. In summary, the present study aims to examine the effects of lifestyle and the number of children on the relationship between widowhood and life satisfaction among elderly adults using a moderated mediation model. Lifestyle is hypothesized to mediate the relationship between widowhood and life satisfaction, whereas the number of children further moderates the relationships between widowhood and lifestyle and between lifestyle and life satisfaction. National survey data were adopted to fit the proposed model. From a theoretical point of view, the present study fills a research gap by examining the indirect effects of widowhood on life satisfaction via lifestyle among elderly adults. Moreover, this study could provide implications regarding China's two-child policy whether the increase of the number of children could decrease the negative impacts of widowhood on life satisfaction.

## Methods

### Participants and Procedures

The 2013 CGSS, which was adopted for the current study, is a large and comprehensive national investigation conducted on the societal, community, family, and individual levels (http://www.chinagss.org). The CGSS adopted a multistage, stratified random sampling design to ensure representativeness. The 2013 CGSS had a proposed sample size of 12,000 individuals and a final sample of 11,438 participants (5,756 men; 5,682 women) with a mean age of 48.60 years (*SD* = 16.39) from all provinces in mainland China, except for Hainan Province and the Xinjiang Uygur Autonomous Region. All data were collected via face-to-face interviews. Written informed consent was obtained from all respondents. Data were published on the CGSS website on January 1, 2015. According to previous studies (35, 36), individuals aged 60 or elderly were selected. After screening for outliers and data with missing values on key variables, such as life satisfaction, number of children, lifestyle, and widowhood, the final sample was with 2,968 participants. No significant gender difference (*t* = −0.041, *p* = 0.934) or age difference (*t* = −0.312, *p* = 0.861) was observed between the included and excluded participants.

### Measures

#### Widowhood and Number of Children

The respondents were asked to self-identify their marital status as (a) first marriage with a spouse; (b) remarried with a spouse; or (c) widowed. According to Jiao's (37) study of the first two groups, which were classified as non-widowed, the independent variable of widowhood (i.e., widowed or non-widowed) was operationalized as a binary variable (0 = *non-widowed*; 1 = *widowed*). In addition, the participants were asked to report the number of children they had, ranging from 1 to 10.

#### Life Satisfaction

The following item was the only item used to evaluate the participants' perceptions of life satisfaction: “Generally speaking, do you think you are living a happy and satisfying life?.” The participants were asked to rate the item on a 5-point Likert scale that ranged from 1 (*very unhappy*) to 5 (*very happy*). Previous studies indicated that the single-item method was appropriate for measuring happiness and life satisfaction in large national surveys (38).

#### Lifestyle

Lifestyle was evaluated by a 9-item, 3-facet survey; namely, a 2-item social network subscale (i.e., meeting relatives who are not living together and gathering with friends); a 4-item outdoor activity subscale (i.e., shopping, attending cultural activities, taking part in physical exercise, and watching live sports); and a 3-item indoor leisure activity subscale (i.e., reading books, newspapers, and magazines; listening to music at home; and surfing the internet) (39). Each item was paired with a 5-point Likert scale. All participants were asked to rate the activities from 1 (*never*) to 5 (*daily*). The mean scores of the nine items were calculated to indicate the frequency of participating in various lifestyle activities (i.e., joining a social network, participating in indoor or outdoor leisure activities). Confirmatory factor analysis showed acceptable goodness of fit (chi-square = 161.107; CFI = 0.95; TLI = 0.93; RMSEA = 0.052; 90% CI = 0.045, 0.059). The standardized factor loadings ranged from 0.327 to 0.633 with significance at the 0.001 level. The Cronbach's α of the lifestyle scale was 0.655.

### Data Analysis

First, descriptive statistics and Pearson and partial correlations analysis were conducted to report demographic characteristics and relationship between lifestyle, number of children and life satisfaction. To identify whether there were differences between widowed and non-widowed groups in life satisfaction, lifestyle and the number of children, analysis of variance was conducted. After revealing the differences caused by different marital status, hierarchical regressions were then conducted to see to what extent life satisfaction was influenced by the variables. Based on these analyses, a moderated mediation model was then established to reveal the relationship between marital status, the number of children, lifestyle, and life satisfaction.

## Results

### Description and Correlations

Table 1 presents the demographic characteristics of this study. The mean age of 2,968 participants was 69.12 (*SD* = 7.24), with 1,515 (51.0%) men and 1,453 (49.0%) women. A total of 1,351 respondents (54.5%) came from countryside, while 1,617 (45.5%) had urban residence, including 156 (5.3%) individuals used to live in the rural area. Seven hundred sixty-six respondents were widowed (253 males and 513 females) and 2,202 were non-widowed (1262 males and 940 females). Overall, the sample had a median education level of primary school, a median socioeconomic status of average. The mean score of the number of children, life satisfaction and lifestyle were, respectively, 2.82 (*SD* = 1.491), 3.80 (*SD* = 0.858), 1.98 (*SD* = 0.583).

**Table 1:** Sample characteristics.

**Demographic characteristics (***N*** = 2,968)**	**Mean (***SD***)/***N*** (%)**
Age	69.12 (7.238)
**Gender**	
Male	1,515 (51%)
Female	1,453 (49%)
**Residence**	
Urban	1,351 (45.5%)
Rural	1,617 (54.5%)
**Martial status**	
Widowed	766 (25.8%)
Male	253 (33.0%)
Female	513 (67.0%)
Non-widowed	2,202 (74.2%)
Male	1,262 (57.3%)
Female	9.40 (42.7%)
Educational level	1.57 (0.940)
Socioeconomic state level	2.61 (0.720)
Number of children	2.82 (1.491)

Table 2 lists the results of the Pearson and partial correlations. The results indicate positive correlations between lifestyle and life satisfaction (*r* = 0.17, *p* < 0.01). The number of children had a positive correlation with life satisfaction (*r* = 0.04, *p* < 0.05) but was negatively related to lifestyle (*r* = −0.22, *p* < 0.01). After controlling for demographic variables including gender, age, location, education, and socioeconomic status, the size of these correlations declined but was still significant. Thus, the demographic variables were not controlled in the following analyses.

**Table 2:** Pearson and partial correlations.

**Variable**	**Pearson correlations (Partial correlations)**		
	**1**	**2**	**3**
1. Lifestyle			
2. Satisfaction with life	0.17** (0.12**)		
3. Number of children	−0.22** (−0.05**)	0.04* (0.04*)	
*M*	1.98	3.80	2.82
Median	1.89	4.00	3.00
*SD*	0.58	0.86	1.49

*The controlled variables were gender, age, education, socioeconomic status, and location*.

* *p < 0.05*,

** *p < 0.01*.

### Analysis of Variance

Analysis of variance was conducted to reveal the differences between widowed and non-widowed participants. The results indicate significant differences in life satisfaction (*F* = 5.429, *p* < 0.05), lifestyle (*F* = 33.731, *p* < 0.001), and the number of children (*F* = 82.523, *p* < 0.001) between the two groups. Specifically, the life satisfaction in the non-widowed group (*M* = 3.820, *SD* = 0.834) was higher than that of the widowed group (*M* = 3.73, *SD* = 0.919). The frequency of participating in various lifestyle activities was significantly higher among non-widowed elderly individuals (*M* = 2.015, *SD* = 0.592) than in widowed individuals (*M* = 1.874, *SD* = 0.543). The number of children was significantly lower among nonwidowed elders (*M* = 2.67, *SD* = 1.406) than among widowed elders (*M* = 3.23, *SD* = 1.643).

### Hierarchical Regressions

Hierarchical regressions were conducted using life satisfaction as the dependent variable. Table 3 indicates that all four regression equations were statistically significant (*F* > 4.438, *p* < 0.05). Collinearity statistics indicated that all variance inflation factor (VIF) values were <5.0. This finding indicates that the multicollinearity of demographic factors, widowhood, lifestyle, life satisfaction, and the number of children is not an issue in our data. In the first step, demographic factors including gender, age, education, socioeconomic status, and location explained 9.4% of the variance of life satisfaction, with the effect size of 0.104. In the second step, widowhood (*t* = −2.271, *p* < 0.05) explained 0.2% of the variance of life satisfaction. Then in the third step, lifestyle (*t* = 6.660, *p* < 0.01) additionally contributed 1.3% of the variance and widowhood became significant. In the last step, the number of children (*t* = 2.451, *p* < 0.05) contributed 0.2% of the variance of life satisfaction. These contributors explained 11.1% of the variance in life satisfaction among elderly individuals, with the effect size of 0.125.

**Table 3:** Hierarchical regression of demographic variables, marital status, lifestyle, and number of children.

**Independent variables**	**Dependent Variable: Life satisfaction**															
	**Step 1**				**Step 2**				**Step 3**				**Step 4**			
	***b***	**β**	***t***	**VIF**	***b***	**β**	***t***	**VIF**	***b***	**β**	***t***	**VIF**	***b***	**β**	***t***	**VIF**
Age	0.006	0.048	2.748*	1.014	0.007	0.062	3.321**	1.123	0.008	0.071	3.861**	1.131	0.006	0.051	2.556*	1.347
Gender	0.075	0.044	2.431*	1.048	0.089	0.052	2.842*	1.092	0.090	−0.053	2.907*	1.092	0.086	0.050	2.747*	1.097
Residence	−0.009	0.010	−0.484	1.324	−0.009	−0.010	−0.488	1.324	−0.042	−0.047	−2.279*	1.429	−0.033	−0.037	−1.737*	1.491
Socioeconomic status	0.365	0.306	16.947**	1.067	0.361	0.304	16.781**	1.071	−0.037	−0.041	−1.946**	1.481	−0.032	−0.035	−1.630**	1.504
Education	−0.003	0.019	−1.37	1.383	−0.006	−0.007	−0.327	1.393	0.337	0.283	15.564	1.102	0.334	0.281	15.400	1.106
Marital status					−0.085	−0.043	−2.271*	1.186	−0.082	−0.042	−2.204*	1.186	−0.081	−0.041	−2.196*	1.186
lifestyle									0.199	0.135	6.660**	1.375	0.203	0.138	6.773**	1.378
Number of children													0.029	0.050	2.451*	1.361
*R* ^2^	0.094				0.096				0.109				0.111			
*F*	61.452**				52.142**				51.683**				46.050**			
Δ*R*^2^					0.002				0.013				0.002			
Δ*F*					5.159*				44.351**				6.009*			

*Beta means beta densities and VIF means variance inflation factor*.

* *p < 0.05*;

** *p < 0.01*.

### Moderated Mediation Model

A moderated mediation model was established using the bias-corrected bootstrap confidence interval method (bootstrap samples = 5,000). Data were analyzed by the PROCESS macro in SPSS (Mode 59) using a path analysis method (40). According to Hayes (40), PROCESS is a computational procedure that can implement moderated mediation analysis in integrated conditional process models. The regression coefficients listed in Table 4 indicate that lifestyle mediated the relationship between widowhood and life satisfaction (b_1_ = 0.129, *p* < 0.05; c_1_' = −0.206, *p* = 0.093). China's One-Child Policy was proved to be an exogenous source of variation in the number of children which also influenced by mothers' age. Therefore, age was set as control variable so as to reveal the true influence caused by the number of children toward lifestyle and life satisfaction (41). When lifestyle was set as the consequent, the interaction between widowhood and the number of children was significant and positive (a_3_ = 0.053, *t* = 3.512, *p* < 0.001, CI = 0.024, 0.083). By contrast, the analysis showed that widowhood has a negative effect (a_1_ = −0.264, *t* = −5.021, *p* < 0.001) on lifestyle. The number of children negatively moderated the negative effects of widowhood on lifestyle. Since the interaction between lifestyle and the number of children was significant and positive, the number of children negatively moderated the negative influence of widowhood on life satisfaction via lifestyle. These results indicated that the elderly with more children were more likely to connect with their social networks and participate in indoor and outdoor activities after losing their spouse, leading to a higher level of life satisfaction. Moreover, the results also indicated that as the number of children increased, the degrees of direct (i.e., negative effects of widowhood on life satisfaction) and indirect (i.e., negative effects of widowhood on life satisfaction via lifestyle) effects declined. Specifically, the indirect effects of widowhood on life satisfaction through lifestyle were significant and positive at low levels (95% bootstrap CI = −0.062, −0.021) and moderate levels (95% bootstrap CI = −0.048, −0.018) of number of children, but became non-significant at high levels (95% bootstrap CI = −0.033, 0.007). This finding indicated that the fluctuations of life satisfaction caused by widowhood are less likely to be explained by discrepancies in lifestyle when elderly individuals have more than four children.

**Table 4:** Results of moderated mediation analysis.

		**Consequent**						
		**lifestyle**		**Life satisfaction**				
**Antecedent**		**coefficient**	**SE**	***p***		**Coefficient**	**SE**	***p***
Marital status	a_1_	−0.264	0.053	<0.001	c_1_'	−0.206	0.079	0.093
Lifestyle		–	–	–	b_1_	0.129	0.056	<0.05
Number of children	a_2_	−0.100	0.009	<0.001	c_2_'	−0.070	0.040	0.077
Marital status × number of children	a_3_	0.053	0.015	<0.001	c_3_'	0.037	0.023	0.105
Lifestyle × number of children	–	–	–	–	b_2_	0.054	0.019	<0.01
Age (control variable)		0.002	0.002	<0.01		0.004	0.002	0.103
Constant		2.183	0.106	<0.001		3.197	0.194	<0.001
		*R*^2^ = 0.057				*R*^2^ = 0.040		
		*F*_(4,2,963)_ = 44.638, *p* <0.001				*F*_(6,2,961)_ = 20.522, *p* <0.001		
		**Moderator level**	**Effect**			***SE***	***p***	
Direct effect at values of the moderator		1.328	−0.157			0.054	<0.01	
		2.819	−0.102			0.038	<0.01	
		4.310	−0.047			0.047	0.311	
		**Moderator level**	**Effect**			**Boot** ***SE***	**95% Bootstrap CI**	
Indirect effect at values of the moderator		1.328	−0.039			0.011	−0.062, −0.021	
		2.820	−0.032	0.008			−0.048, −0.018	
		4.310	−0.013	0.010			−0.033, 0.007	

## Discussion

Using data from a national survey, the present study revealed that widowed elderly individuals were less likely to be satisfied with their lives and participate in social networks, indoor leisure activities, and outdoor activities compared with non-widowed elderly individuals. The results of the moderated mediation model demonstrated that lifestyle partly mediated the relationship between widowhood and life satisfaction. The degree of the negative effects of widowhood on lifestyle declined as the number of children increased. The negative influences of widowhood on life satisfaction through lifestyle were significant at low (i.e., one child) and moderate levels (i.e., two or three children) and nonsignificant at higher levels (i.e., four or more children). These results indicated that the influence of widowhood on life satisfaction can be explained by lifestyle differences between widowed elderly individuals and non-widowed elderly individuals. In addition, elderly individuals with more children—up to two or three—experience higher life satisfaction and more colorful lifestyles after spousal loss.

The discrepancy of life satisfaction between widowed elderly individuals and non-widowed elderly individuals can be attributed to the existence of “significant others,” such as a spouse (42, 43). Specifically, under the framework of cognitive theory and social interaction theory, individuals who feel lonely and less satisfied are those who experience deficiencies in their relationships with their significant others (44–46). Previous research showed that loneliness is likely to cause depression if the emptiness in one's social life or emotional desires cannot be satisfied (47). Therefore, the loss of a significant other (e.g., spouse) tends to narrow social networks and causes damage to their social relationships; this situation offers less social support and spiritual consolation to widowed elderly adults, which negatively influences their life satisfaction (48). For example, quality of life, health, and self-perception can all be affected by spouses (21). By contrast, those who live with their spouses tend to show the lowest levels of loneliness (49).

As discussed, the lifestyles of elderly individuals were deeply influenced by their spouses (39, 50), who are the significant others in their lives. According to Hagedoorn et al. (51), the social resources of the elder linked with their spouse became increasingly important, which means that in some cases, an individual's spouse might have been their source of motivation to stay engaged and take part in various lifestyle activities. Moreover, elderly individuals might be more comfortable engaging in activities with their spouse rather than by themselves. Therefore, widowhood, to some extent, prevents elderly people from being motivated to participate in variety lifestyle activities, during which social connections could be built to reduce loneliness (52). Research also showed that after a marriage dissolves, elderly individuals would join social networks and participate in indoor and outdoor activities less frequently (53). In this way, social resources and social relationship could be inaccessible for widowed elderly individuals. Thus, it is comparatively hard for widowed elderly people to achieve life satisfaction due to the limited social relationships with significant others (i.e., spouses) (54). These discussions were consistent with our findings that lifestyle can mediate the relationship between widowhood and life satisfaction. This indicated that the difference in life satisfaction between widowed and married elderly individuals can be explained by distinctions in their lifestyles.

However, according to the mechanism of selective optimization with compensation, activities and relationships are reviewed and prioritized selectively so that by the use of compensatory behaviors, experiences can be optimized and well-being maintained (55). The well-documented (56, 57) continuity theory suggests that aging individuals tend to maintain similar social relationships, roles, and lifestyles compared with those in their early years; they also seek stability throughout the course of their lives. Specifically, for individuals who have experienced widowhood, being a parent of their adult children serves a role of continuity, providing them easy access to substitutional activities. Motivated by seeking stability and continuity in their later years, widowed individuals tend to get involved in child-related activities, which in turn, enrich their lifestyle and reduce the harmful effects of widowhood on social participation.

Having children can influence the lifestyles of elderly individuals in various ways. First, parenting and filial responsibilities are two core aspects in Chinese culture (58–60). When parents age, their children are obliged to care for them; thus, elderly individuals who have lost their spouse tend to live with or close to their children (61). This culture strengthens their connections and enhances the probability of mutual influence. Second, given the rapid economic and social transition in recent years, the intergenerational cultural transmission in Chinese families is commonly predominated by young individuals (62); anthropologist Margaret Mead called this concept the prefigurative culture (63). Therefore, the more children elderly individuals have, the more likely their lifestyles will be affected by their children as the frequent use of the internet and social media (64) and engaging in modern lifestyle activities (e.g., going shopping, watching live sports, and attending cultural events) to blend in with the younger generations (30). Moreover, being parents generally means having broader social networks. Assuming the social role of parenthood prompts them to engage more in community or service-oriented activities, make more connections with extended kin, and change their social lives by avoiding unhealthy lifestyles and risky behaviors (65, 66).

The mediating effects of the number of children on the relationship between lifestyle and life satisfaction, having more social interactions, and living with adult children result in improved life satisfaction (67). Many studies showed that adult children provide instrumental and emotional support for their parents, which have positive impacts on the life satisfaction of elderly individuals (68) and moderate the negative effects of widowhood on their life satisfaction (69).

In accordance with traditional Chinese culture, living in a big family is the life goal of many elderly individuals, and engaging in family-related social activities provides them with a sense of belongingness (70). In continuity theory, the continuity of elderly individuals' life goals and family roles, shown in family-related social activities, is a social integration process that increases their life satisfaction in a cultural context.

However, in some cases, more does not always mean merrier. Our result demonstrates that the negative impacts of widowhood on life satisfaction decreases when the number of children increases from one to three and the moderation effect reaches a significant level. However, for individuals with four or more children, these effects lose their statistical significance. This phenomenon suggests that the buffering effects only occur if the number of children is no more than three. In this case, having children can help elderly parents compensate for the loss of social resources and disengagement in activities caused by widowhood, thereby enhancing their life satisfaction by enriching their lifestyle. However, the positive effects vanish as the number of children increases due to the following reasons. First, intergenerational conflicts and financial arguments are likely to occur, and the children might shirk their filial responsibilities or shift the burden of care to other siblings as the number of children increases (71). Second, the positive impacts of their children are also associated with the quality of their children. More educated children can bring about more material support and health care services for elderly parents (72), but having more children may dilute family resources; this reduces the human capital investment in each child and impairs the quality of children, according to resource dilution theory in sociology (73, 74). Therefore, having four or more children might not positively affect the life satisfaction of the widowed individuals.

The limitations of this study should be acknowledged. First, the life satisfaction was operationalized with a single-item measure in this study due to the limitations of the data. Future research should adopt more solid and comprehensive scales, such as the Flourishing Scale and Satisfaction with Life Scale. Second, we adopted self-reported, subjective measures of widowhood and lifestyle. However, the ability of this scale to reflect an individual's marital status, participation in social networks, and outdoor and indoor activities should be discussed further. Tracking observations or recording lifestyle using an objective approach can improve the reliability and validity of future research. Third, this study only considered the number of children as a moderator, whereas other factors such, as the rural or urban setting, might also act as a moderator. By including more possible variables in the model, future studies might enhance the present understanding of life satisfaction among elderly individuals and the other 88.9% of the variance in life satisfaction among elderly individuals could be explained further. Despite these limitations, our findings are generally valuable because they explicitly indicate the mediating effects of lifestyle in the relationship between widowhood and life satisfaction in groups of elderly individuals. The moderator role of the number of children is also demonstrated in this study based on this finding.

## Data Availability Statement

Publicly available datasets were analyzed in this study. This data can be found at: http://cnsda.ruc.edu.cn/index.php?r=projects/view&amp;id=93281139.

## Author Contributions

WD designed the study, supervised, and administrated the project. CY wrote the manuscript and prepared the submission materials. XS revised the manuscript and conducted the additional analyses. All authors contributed to the article and approved it for publication.

## Funding

Funding was provided by the Philosophy and Social Sciences Major Theoretical and Practical Issues Research Project of Shaanxi Province Study on the Impact of Youth Social Isolation on Health (2021ND0190) and the National Social Science Foundation Major Project Research on Optimization of Chinese Healthy Aging Service System in the New Era (21ZDA103).

## Conflict of Interest

The authors declare that the research was conducted in the absence of any commercial or financial relationships that could be construed as a potential conflict of interest.

## Publisher's Note

All claims expressed in this article are solely those of the authors and do not necessarily represent those of their affiliated organizations, or those of the publisher, the editors and the reviewers. Any product that may be evaluated in this article, or claim that may be made by its manufacturer, is not guaranteed or endorsed by the publisher.
